# Supplementary material for: Combined genetic and chemical methods boost the precision of tracing illegal timber in Central Africa
Source: Commun Earth Environ. 2025 Oct 15;6(1):789. doi: 10.1038/s43247-025-02698-z (PMC12527923; doi:10.1038/s43247-025-02698-z)
Supplement: Supplementary file 2 — Supplementary information [file 43247_2025_2698_MOESM2_ESM.pdf]

Supplementary Information

Supplementary Methods
S1 Modified DNA isolation protocol

Extraction of DNA with CTAB

Necessary buffers:

- NaCl 5 M: 29,22 g for 100 mL - EDTA 0.5 M pH8.0: 18,61 g EDTA + 2 g NaOH per 100 mL (to adjust pH)
- Tris - HCl 1 M pH8.0: 8.88 g TrisHCl + 5.3 g Tris Base for 100 mL (to adjust pH)
- Cold Isopropanol (-20 ° C)
- Cold 70% ethanol (-20 ° C)
- Ethanol 95% cold (-20 ° C)
- Chloroform : isoamylalcohol (24 : 1)
- RNase A 20 mg/mL
- TE pH 8.0: 1 mL Tris 1 M pH8.0 + 200 µl of EDTA 0.5 M pH8.0 to 100 mL
- H2O milliQ

For 25 mL CTAB buffer:

Volume/quantity to	Final concentration
0.5 g CTAB	2% CTAB
7 mL of NaCl 5 M	1.4 M NaCl
1 mL of EDTA 0.5 M	20 mM EDTA pH 8.0
2.5mL of 1 M Tris - HCl	100 mM Tris - HCl pH 8.0
**Storage at room temperature**	
1 mL DTT	2% DTT
1 g PVP	4% PVP
0.5 mL Proteinase K (20 mg/mL)	0.**4** mg/mL Proteinase K

Protocol:

Weigh approximately 100 mg of powder in a 2 mL tube.

1. Prepare a master mix with 22.560 mL CTBA buffer (with PVP) + 960 mL DTT + 480 µL Proteinase K (20 mg/mL). OR 40µl of DTT and 20µl of Proteinase K per sample/tube.
2. Add 900 µl of CTAB buffer pre-heated at 60 ° C. Incubate at 60 °C for 1 hour stirring occasionally (every 10 min).
3. Add chloroform : isoamylalcohol (24 : 1) 1V or 1 mL. **(900 µl)** Mix 1 min by inverting the tubes. Centrifuge 10 min at 14 000 x g. Retrieve the upper aqueous phase and transfer it into a clean 2 mL tube.
4. Add 10 µL of RNAse A (20 mg/ml). Incubate 1 hour h at 37 ° C.
5. Add chloroform : isoamylalcohol (24 : 1) 1V or 1 mL. Mix 1 min by inverting the tubes. Centrifuge 10 min 14 000 x g. retrieve the upper aqueous phase and transfer it into a clean tube of 2 mL.
6. Estimate the amount recovered. Add 2/3 volume of cold isopropanol. Leave ½ hour minimum at 20ºC. Centrifuge at max speed max for 15 min at 4ºC.
7. Remove the supernatant. Add 700 µL of EtOH 70% cold. Invert the tube several times to loosen the pellet. Centrifuge at max speed for 5 min at 4°C.
8. Remove the supernatant. Add 700 µL of EtOH 95% cold. Invert the tube several times to loosen the pellet. Centrifuge at max speed for 5 min at 4°C. 9. Dry the pellet. Resume in 100 µL H_2O milliQ (or TE^{-4}).

Table S1: **Mean and standard deviation of the three isotope ratios**, averaged within sites as well as across sites, expressed relative to the respective international standards in ‰.

	$\delta^{18}O$	$\delta^{2}H$	$\delta^{34}S$
Mean of the averages within sites	28.0	14.4	7.2
SD within sites	0.7	7.7	0.8
Mean across all sites	27.9	12.7	7.2
SD across sites	1.1	10.7	1.4

Table S2: **Number of trees analyzed per tracing method per site.** Number of overlapping trees used for test and training datasets is depicted in Figure S1. EL = multi-element analysis, SNP = pSNPs.

	EL	SNP	^{34}S	^{2}H	^{18}O
CAM1	16	16	2	7	7
CAM2	19	19	4	6	9
CAM3	15	15	4	4	7
CAM4	18	18	4	10	10
CAM5	20	20	4	6	9
CAM6	19	19	3	9	9
CAM7	19	19	3	5	9
CON2	16	16	2	7	6
CON4	19	19	4	6	6
CON7	18	18	4	6	6
CON8	18	18	4	6	6
GAB1	17	17	4	9	6
GAB5	20	20	4	9	6
Total	234	234	46	90	96

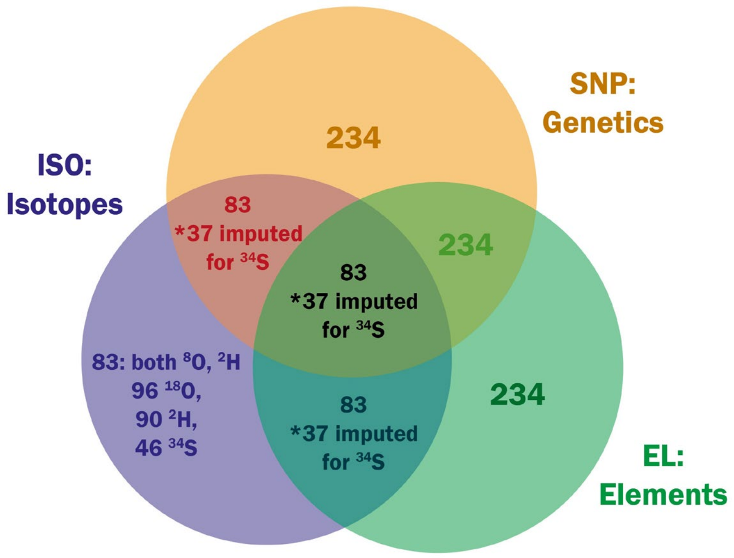

Figure S1: **The overlap in number of trees included per tracing method.** Venn diagram where each circle depicts the number of measured trees per method and the overlap between the circles depicts the number of trees for which multiple methods have been applied. Only the trees for which genetic data, elemental data as well as δ^2H and δ^{18}O data was available were used as test trees for validating the models (83 trees in the center). 37 of those 83 did not have measured δ^{34}S, we imputed those with site means. For the training sets for model development, all 234 trees (minus the 41 test trees each cycle) were included and across the three isotopes, missing data was imputed with site means. Please note that not all δ^{18}O and δ^2H measurements overlapped: there were 83 trees for which both were measured.

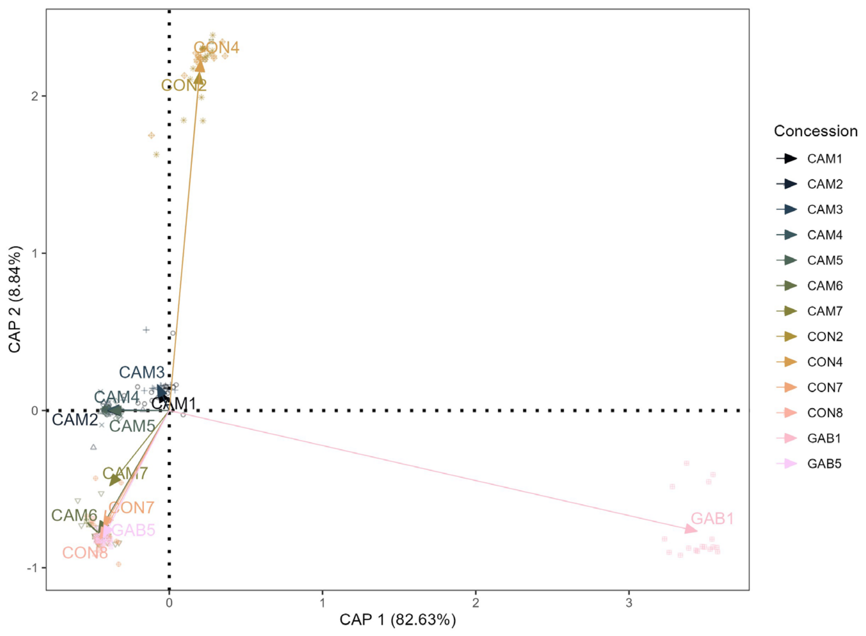

Figure S2: **Db-RDA analysis of the genetic distances in the pDNA**, calculated based on 1-proportion of shared alleles across 13 sites, using 238 pDNA loci for 234 trees. The colored dots indicate the trees, colored by site.

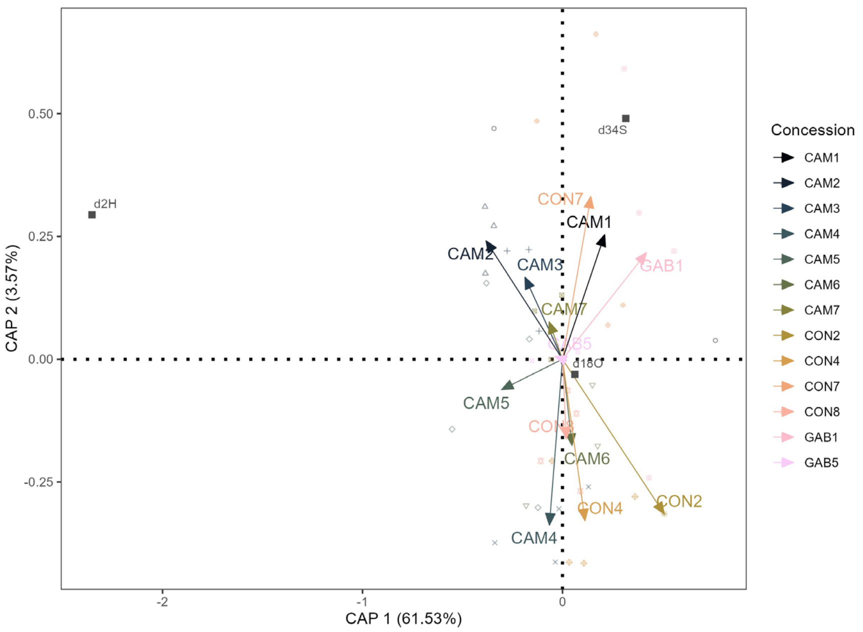

Figure S3: **Db-RDA analysis of isotopic composition** of the 46 trees across 13 sites in which all three isotope ratios were measured (d18O, d2H and d34S), based on Chord distances. The colored dots indicate the trees, colored by site. The grey dots indicate the elements.

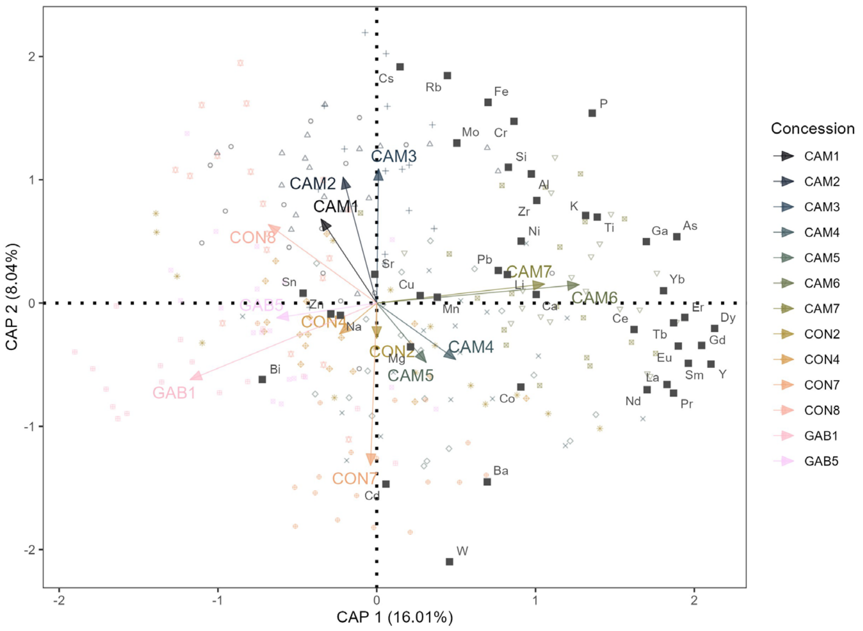

Figure S4: **Db-RDA analysis of the multi-elemental composition** of 234 trees across 13 sites, based on Chord distances. The colored dots indicate the trees, colored by site. The grey dots indicate the elements.

Figure S5: Distance to predicted origin (km). Depicted as % of the total number of test trees per distance bin per method. The lowest bar represents correct site origin assignment, which is the identification accuracy, with error bar that indicates the variation (st. dev.) in identification accuracy, estimated using different test and training datasets of the Random Forest models. SNP = pSNPs, ISO = stable isotope ratios, EL = multi-element analysis, ALL = all three methods, RDM = the occurrence of pairwise distances in the reference dataset, representing the expected result of fully random assignments. This is a different representation of Figure 3 where each bar is stacked and standard deviation is depicted only for the first distance class.

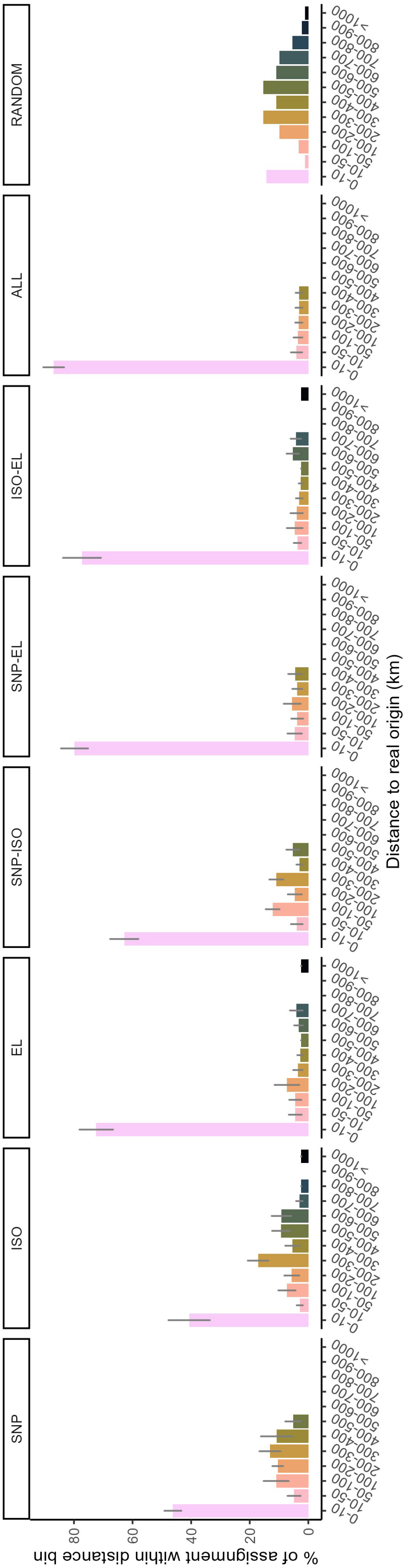

Figure S5: **Distance to predicted origin (km).** Depicted as % of the total number of test trees per distance bin per method. The lowest bar represents correct site origin assignment, which is the identification accuracy, with error bar that indicates the variation (st. dev.) in identification accuracy, estimated using different test and training datasets of the Random Forest models. SNP = pSNPs, ISO = stable isotope ratios, EL = multi-element analysis, ALL = all three methods, RDM = the occurrence of pairwise distances in the reference dataset, representing the expected result of fully random assignments. This is a different representation of Figure 3 where each bar is stacked and standard deviation is depicted only for the first distance class.
